# KRIBB11 Induces Apoptosis in A172 Glioblastoma Cells via MULE-Dependent Degradation of MCL-1

**DOI:** 10.3390/molecules26144165

**Published:** 2021-07-08

**Authors:** Kyunghyun Yoo, Hye-Hyeon Yun, Soon-Young Jung, Jeong-Hwa Lee

**Affiliations:** 1 Department of Biochemistry, College of Medicine, The Catholic University of Korea, Seoul 16591, Korea; ted13579@hanmail.net (K.Y.); nice1205@hanmail.net (H.-H.Y.); syjjeong@hanmail.net (S.-Y.J.); 2 Institute for Aging and Metabolic Diseases, College of Medicine, The Catholic University of Korea, Seoul 16591, Korea; 3 Department of Biomedicine & Health Sciences, Graduate School, College of Medicine, The Catholic University of Korea, Seoul 16591, Korea

**Keywords:** glioblastoma, KRIBB11, MCL-1, apoptosis

## Abstract

KRIBB11, an HSF1 inhibitor, was shown to sensitize various types of cancer cells to treatment with several anticancer drugs. However, the exclusive effects of KRIBB11 in preventing the growth of glioblastoma cells and the related mechanisms have not been elucidated yet. Herein, we aimed to examine the potential of KRIBB11 as an anticancer agent for glioblastoma. Using MTT and colony formation assays and Western blotting for c-PARP, we demonstrated that KRIBB11 substantially inhibits the growth of A172 glioma cells by inducing apoptosis. At the molecular level, KRIBB11 decreased anti-apoptotic protein MCL-1 levels, which was attributable to the increase in MULE ubiquitin ligase levels. However, the constitutive activity of HSF1 in A172 cells was not influenced by the exclusive treatment with KRIBB11. Additionally, based on cycloheximide chase assay, we found that KRIBB11 markedly retarded the degradation of MULE. In conclusion, stabilization of MULE upon KRIBB11 treatment is apparently an essential step for degradation of MCL-1 and the subsequent induction of apoptosis in A172 cells. Our results have expanded the knowledge on molecular pathways controlled by KRIBB11 and could be potentially effective for developing an inhibitory therapeutic strategy for glioblastoma.

## 1. Introduction

Glioblastoma multiforme (GBM) is the most aggressive and frequently occurring primary brain tumor with a median survival of less than 1 year [1]. The current optimal treatment for GBM is based on a combination therapeutic strategy that includes surgical resection, followed by radiation and chemotherapy [2]. However, the intrinsic characteristics of GBM cells, which include rapid growth and infiltration into the normal brain tissues, hinder the successive removal of the primary lesion; it frequently leads to postoperative recurrence despite the advances in intraoperative imaging techniques that facilitate intraoperative analysis of tumor and normal brain tissues [3,4]. In addition, the emergence of chemo resistance, especially to temozolomide (TMZ), which is the most effective drug for GBM at present, further contributes to tumor recurrence, resulting in poor clinical outcomes [5]. Therefore, it is urgent to develop a new and effective drug for GBM based on a molecular mechanism that is different from that of TMZ.

KRIBB11 (*N2*-(1*H*-indazole-5-yl)-*N6*-methyl-3-nitropyridine-2,6-diamine) has been identified as an HSF (heat shock factor)1 inhibitor, based on screening of a chemical library using heat shock-dependent luciferase reporter constructs, in which four copies of palindromic HSE (heat shock element) had been inserted [6]. Subsequent studies demonstrated that KRIBB11 sensitizes colorectal cancer cells upon apoptosis induced by a HSP90 inhibitor or by benzimidazole carbamate [7,8]. In addition, the combined treatment with an AKT inhibitor and KRIBB11 exhibited synergistic effects in the suppression of breast cancer cell growth of different subtypes in vitro and in vivo [9]. Through suppression of mevalonate- and cholesterol biosynthesis-related gene expression, KRIBB11 sensitized antiproliferative effects of simvastatin in several hepatocellular carcinoma cell lines [10]. A probable molecular basis for KRIBB11 priming cancer cells to apoptosis could include reduced expression of HSF1 target genes; this is apparently a potential mechanism, because the sensitizing effects of KRIBB11 were reproduced upon HSF1 silencing [7,8,9]. Considering that the high expression of HSF1 in various types of cancers are frequently associated with poor prognosis and resistance for chemotherapy [11], the sensitizing effects of KRIBB11 on the suppression of cancer cell growth appear to be a promising therapeutic strategy for clinical application.

In glioma cells, KRIBB11 reactivates apoptosis after combined treatment with BH3 mimetics, such as AT101 and ABT-737 [12]. In addition to the apoptosis-priming effect, treatment with KRIBB11 alone substantially induced apoptosis in U251 glioma cells as determined by several parameters including Annexin-V staining and caspase activity. However, the mechanism by which KRIBB11 exclusively induces apoptosis has not been elucidated in detail, although its pro-apoptotic activity can be attributed to the modulation of HSF1 activity. In the present study, the antitumor activity of KRIBB11 was exclusively examined with respect to another glioma cell line, A172 cells. We observed that KRIBB11-induced apoptosis was accompanied by a significant decrease in the levels of anti-apoptotic protein MCL-1 without alteration in the expression of HSF1-target proteins. Our results indicate that MCL-1 degradation in an HSF1-independent manner is an essential step for the modulation of apoptotic cell death brought about by KRIBB11 in glioma cells.

## 2. Results

### 2.1. KRIBB11 Induces Apoptotic Cell Death in A172 Cells

**Figure 1:**
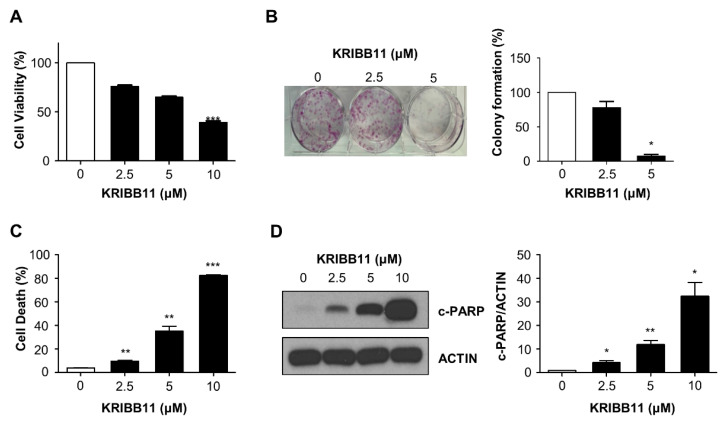
Effect of KRIBB11 on the survival of A172 cells. (**A**) A172 cells were treated with an increasing dose of KRIBB11 for 72 h, and the relative survival was determined using MTT assay. Data was presented as mean ± SE of triplicate experiments. (**B**) Colony forming assay was performed with 1000 cells/well of A172 cells treated with 0, 2.5, or 5 μM of KRIBB11. After 15 days, the colony number was counted with crystal violet staining (**left**). The mean value from three independent experiments were provided as percentage of control (**right**). (**C**) KRIBB11-induced cell death was represented as percentage of PI positive cells from total cells. Values are mean ± SE of three independent experiments. (**D**) Western blotting for cleaved PARP (c-PARP) was performed with lysates from A172 cells treated with various concentrations of KRIBB11 (**left**). Induction of apoptosis was evaluated by quantitation of c-PARP levels from three independent experiments with densitometric analysis after normalizing to β-Actin using Image J software. Data are mean ± SE of three independent experiments (**right**). * *p* < 0.05, ** *p* < 0.01, and *** *p* < 0.001 with respect to control values.

To explore the anti-tumor potential of KRIBB11 in glioblastoma, A172 cells were treated with various concentrations of KRIBB11, and then, the cell survival was evaluated based on several methods. MTT assay indicated that the relative cell survival of A172 cells was inhibited by KRIBB11 in a dose-dependent manner (Figure 1A). The anti-proliferative activity of KRIBB11 was also observed in the colony formation assay, which showed that the colony forming ability of A172 cells treated with 2.5 and 5 μM of KRIBB11 was 78 and 7%, respectively, in comparison to that of the control cells (Figure 1B). In addition, the dead cell proportions of A172 cells notably increased with the increasing dose of KRIBB11 as determined by propidium iodide (PI) staining (Figure 1C). Western blotting revealed that the cleaved-PARP levels gradually increased following KRIBB11 treatment, which indicated that KRIBB11 induced apoptosis in A172 cells (Figure 1D).

### 2.2. Downregulation of MCL-1 Is Essential for KRIBB11-Induced Apoptosis

**Figure 2:**
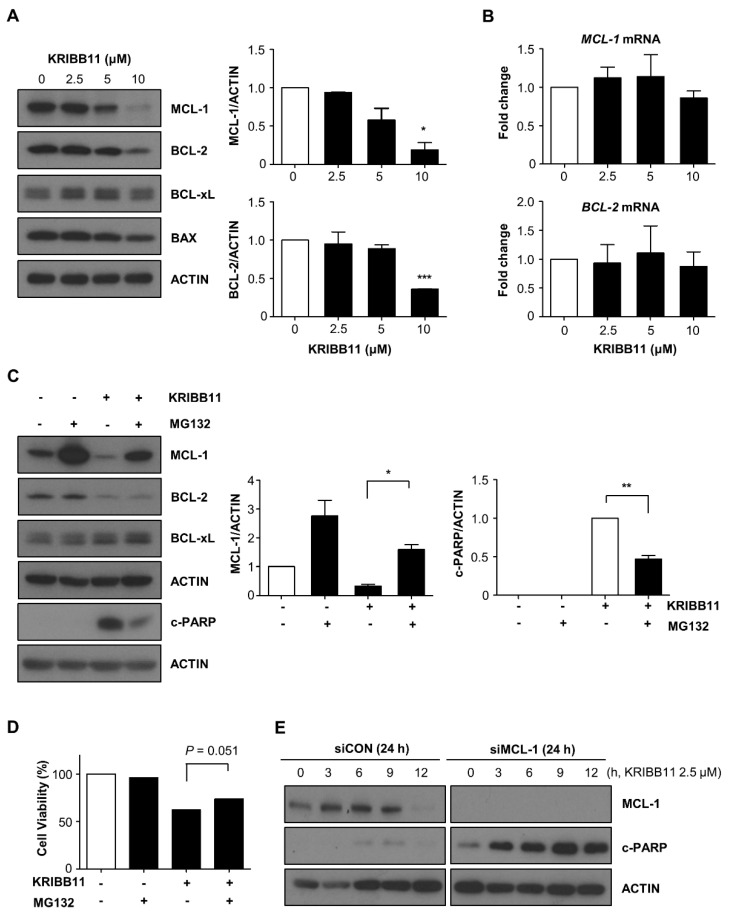
KRIBB11 decreases MCL-1 expression, which was restored by MG132. (**A**) Western blot analysis for expression of MCL-1, BCL-2, BCL-xL, and BAX in A172 cells treated with the indicated dose of KRIBB11 for 48 h (**left**). Quantification of relative levels of MCL-1 or BCL-2 protein were determined by normalizing to β-Actin as in Figure 1 (**right**). (**B**) qRT-PCR analysis for *MCL-1* and *BCL-2* mRNAs after exposure to the indicated dose of KRIBB11. (**C**) Effect of proteasome inhibition was examined using MG132. A172 cells were treated with 10 μM of KRIBB11 for 24 h and subsequently with 10 μM of MG132 for another 4 h followed by Western blotting analysis (**left**). The alteration of MCL-1 and c-PARP levels upon MG132 treatment was presented by densitometric analysis after normalizing to the β-Actin (**right**). (**D**) After treatment as in (**C**), A172 cells were washed with phosphate-buffered saline and then further incubated for 48 h. The relative cell viability was determined by MTT assay. (**E**) Effect of MCL-1 depletion on the KRIBB11-induced apoptosis by transfection 50 nM of MCL-1 specific siRNA and the Western blotting for MCL-1 and c-PARP. Data are presented as means ± SE of three independent experiments. * *p* < 0.05, ** *p* < 0.01, and *** *p* < 0.001 with respect to the control values (**A**) or between indicated groups (**C**).

Subsequently, we examined the change in the expression of BCL-2 family proteins after treatment with KRIBB11 to define the protein responsible for the KRIBB11-induced apoptosis in A172 cells. As shown in Figure 2A, the expression of BCL-2 and MCL-1, which are the two representative anti-apoptotic proteins, decreased following KRIBB11 treatment; however, the expression levels of BCL-xL, another anti-apoptotic protein, remained unchanged. Densitometry analysis indicated that MCL-1 and BCL-2 levels were 19% and 36% relative to the control, respectively, after treatment with 10 μM of KRIBB11 for 48 h. Western blotting also indicated that the expression of pro-apoptotic proteins such as BAX was not notably altered upon exposure to KRIBB11 in A172 cells. To determine if the decrease in MCL-1 and BCL-2 were caused by the loss of protein stability during the apoptotic process, we examined the expression of these proteins in the presence of a caspase inhibitor. As shown in Supplementary Figure S1, the expression of MCL-1 and BCL-2 decreased markedly when cell death was inhibited. Thus, the reduction in the expression of these proteins appeared to be a direct effect of KRIBB11 rather than the result of apoptotic cell death.

Next, we conducted a qRT–PCR analysis to determine if the reduction of MCL-1 and BCL-xL expression was a result of a decrease in their respective mRNA levels. Figure 2B shows that the mRNA expression of MCL-1 and BCL-2 was not significantly suppressed by KRIBB11. Thus, KRIBB11 regulates the translation or stability of MCL-1 and BCL-2 proteins. To test if KRIBB11 impacts MCL-1 or BCL-2 protein stability, we treated A172 cells with MG132, a proteasome inhibitor, following KRIBB11 treatment. As shown in Figure 2C, the decrease in the MCL-1 levels was substantially restored by MG132 treatment; however, BCL-2 expression was not affected by MG132 treatment. Moreover, c-PARP levels that had increased following KRIBB11 treatment were observed to decrease upon MG132 treatment. Thus, the inverse correlation in the MCL-1 and c-PARP levels following KRIBB11 treatment with or without MG132 strongly suggests that the reduction of MCL-1 levels is essential for the KRIBB11-induced apoptotic pathway, which is supported by the partial recovery of relative cell survival (Figure 2D). In line with these results, MCL-1 siRNA further potentiates the anti-apoptotic activity of KRIBB11 as assessed by induction of c-PARP (Figure 2E).

### 2.3. The Constitutive Activity of HSF1 Was Not Affected by KRIBB11 Treatment in A172 Cells

**Figure 3:**
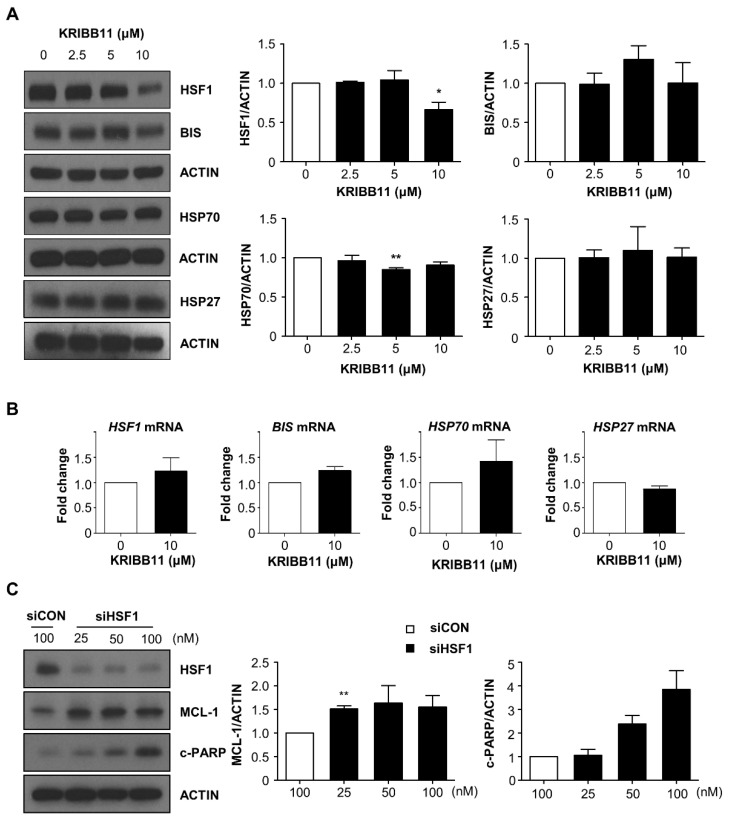
Effect of KRIBB11 on the expression of HSF1 and its target proteins. (**A**) Following treatment with KRIBB11 with the indicated dose, the expression of HSF1 and HSP70 was examined by Western blotting (**left**). Densitometry analysis for HSF1 and HSP70 from three independent experiments was shown in right column. (**B**) The mRNA levels of *HSF1*, *BIS*, *HSP70,* and *HSP27* were determined by qRT–PCR analysis. (**C**) HSF1 expression was suppressed by siRNA (siHSF1) in A172 cells, and the expression of MCL-1 and c-PARP levels were determined by Western blotting analysis (**left**). The relative levels of MCL-1 and c-PARP were determined by densitometry analysis. The value from A172 cells transfected with control siRNA (siCON) was designated as 1.0. Data are mean ± SE of three independent experiments. * *p* < 0.05, and ** *p* < 0.01 vs. control values.

As KRIBB11 has been originally identified as an HSF1 inhibitor [6], we examined if the alteration of HSF1 expression or its activity is involved in KRIBB11-mediated degradation of MCL-1 and apoptosis. Western blot analysis indicated that HSF1 levels decreased upon treatment with high dose of KRIBB11; however, HSP70 levels remained notably unchanged (Figure 3A). qRT–PCR analysis results indicated that the mRNA levels of *HSP70, HSP27,* and *BIS* [13], which are the target genes of HSF1, in A172 cells did not decrease following KRIBB11 treatment (Figure 3B). This indicates that the constitutive HSF1 activity was not suppressed by KRIBB11. HSF1 protein levels decreased following KRIBB11 treatment; however, *HSF1* mRNA levels were not affected by the treatment (Figure 3B). Furthermore, depletion of HSF1 by specific siRNAs increased MCL-1 levels (Figure 3C). Thus, alteration in HSF1 levels or activity was not the cause of reduction in MCL-1 levels upon exposure of A172 cells to KRIBB11.

### 2.4. KRIBB11 Stabilizes Ubiquitin Ligase MULE, Resulting in the Downregulation of MCL-1

**Figure 4:**
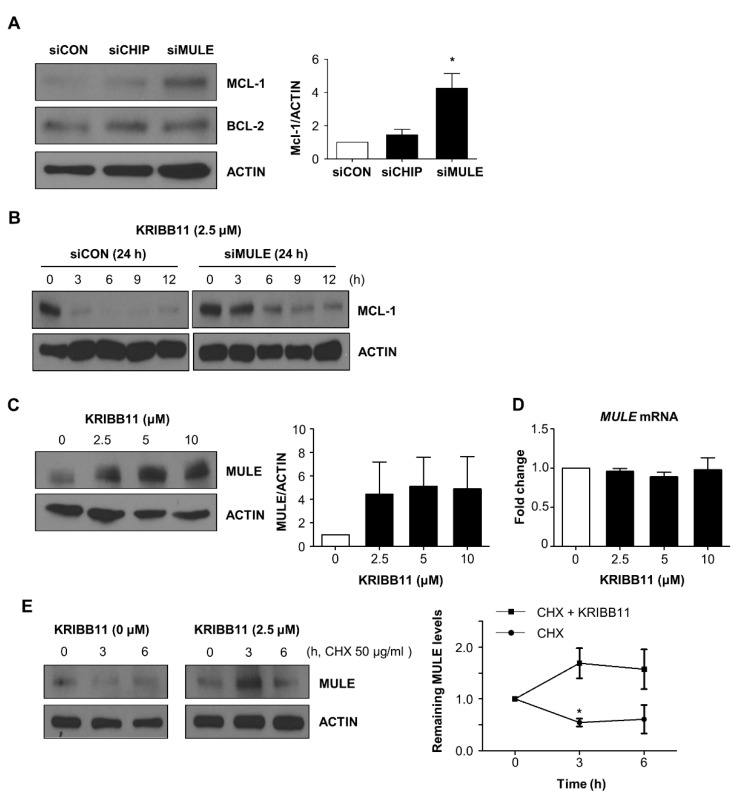
MCL-1 ubiquitin ligase MULE was stabilized by KRIBB11. (**A**) The effect of MULE depletion on MCL-1 was examined by transfection of specific siRNA (100 nM) for 48 h and subsequent Western blotting (**upper**). The relative levels of MCL-1 after depletion of CHIP or MULE were shown by densitometry analysis (**lower**). (**B**) MULE depletion retarded the KRIBB11-induced MCL-1 reduction, which was evaluated by Western blotting. (**C**,**D**). MULE levels after KRIBB11 treatment were determined by Western blotting (**C**) and qRT PCR analysis (**D**). (**E**) Cycloheximide (50 μg/mL) was treated with KRIBB11 (2.5 μM), and the kinetics of MULE expression was compared with cycloheximide only group by Western blotting (**left**). The quantitation of MULE was performed with densitometry (**right**). Data are mean ± SE of three independent experiments. * *p* < 0.05 vs. control values.

Since MCL-1 expression was restored by MG132 treatment, we examined the influence of MCL-1-specific ubiquitin ligase MULE [14,15] on MCL-1 expression in A172 cells. As shown Figure 4A, knockdown of MULE resulted in significant accumulation of MCL-1. The specific effect of MULE on MCL-1 levels was demonstrated by two findings. First, CHIP, another ubiquitin ligase, exhibited no effect on the MCL-1 levels. In addition, BCL-2 expression was not altered by MULE and CHIP. Next, we checked the effect of MULE depletion on MCL-1 degradation induced by KRIBB11. In comparison to the control siRNA treatment, that of MULE siRNA notably delayed the decrease in MCL-1 under the presence of 2.5 μM of KRIBB11 (Figure 4B). This indicates that KRIBB11-mediated degradation of MCL-1 is dependent on MULE. Subsequently, we examined the possibility that KRIBB11 directly affects MULE expression by conducting Western blotting and qRT–PCR analysis. Figure 4C shows that KRIBB11 significantly increased MULE protein levels without notable alterations in mRNA levels under various concentrations of KRIBB11. Thus, the increase in MULE expression after KRIBB11 treatment is apparently associated with the acceleration of MCL-1 decay via the ubiquitin-proteasome pathway. Since mRNA levels of MULE remained unchanged by KRIBB11 treatment, we checked if it is involved in protein stability of MULE using cycloheximide (CHX) chase experiment. Treatment with CHX alone decreased MULE expression to 60% that in the control (the expression at 0 time) at 6 h; however, the co- treatment with KRIBB11 maintained MULE expression at 157% that in the control at the same time point (Figure 4D).

Collectively, the stabilization of MULE ubiquitin ligases upon KRIBB11 treatment apparently accelerates the degradation of MCL-1 and subsequent induction of apoptosis.

## 3. Discussion

Since its recognition as an HSF1 inhibitor, KRIBB11 has been reported to increase susceptibility to various anticancer molecules in different types of cancer cells in vitro [6,7,8,9]. In the present study, we demonstrated that an exclusive treatment with KRIBB11 effectively induces apoptosis in A172 glioma cells through acceleration of MCL-1 degradation, which is not accompanied by any alteration of HSF1 activity. Further, our results revealed that the KRIBB11-induced MCL-1 decrease is attributable to induction of MULE ubiquitin ligase at protein levels, which suggests the involvement of KRIBB11 in the regulation of MULE protein stability. Previously, we observed that KRIBB11 decreases MCL-1 expression in A549 lung adenocarcinoma cells [16]. In addition to confirming the reduction of MCL-1 level by KRIBB11 in other types of cancer cells, such as A172 cells, our present study revealed that the decrease in MCL-1 expression is responsible for the KRIBB11-mediated apoptosis of A172 cells. First, MCL-1 level was restored upon MG132 treatment concomitantly with the decrease in c-PARP levels. Second, MCL-1 depletion by siRNAs further suggests KRIBB11-mediated apoptosis. While our previous study [16] showed MCL-1 is induced upon depletion of MULE, herein, we observed that KRIBB11 stabilized MULE at protein levels based on CHX chase experiments and qRT-PCR analysis, which revealed no increase in MULE mRNA levels. Thus, in conjunction with our previous study, the present results show that KRIBB11 exhibits a novel role in the stabilization of MCL-1 levels, which are critical for determining cell fate, survival, or apoptosis.

KRIBB11 is an inhibitor of HSF1; however, our present study showed no correlation of KRIBB11-mediated reduction of MCL-1 and alteration of HSF1 activity based on the expression of HSF1 target genes including HSP70, HSP27, and BIS, which were not affected by KRIBB11. Moreover, HSF1 depletion did not result in a decrease in MCL-1 levels in A172 cells (Figure 3). These results indicate that the constitutive activity of HSF1 was not involved in KRIBB11-mediated MCL-1 reduction and the subsequent induction of apoptosis in A172 cells. Our findings are inconsistent with those of the previous studies showing that the priming effect of KRIBB11 on anticancer effect of various drugs was mostly reproduced by HSF1 depletion [7,8,9]. One possible explanation for this discrepancy is that the molecular pathway activated exclusively by KRIBB11 might be different from that of KRIBB11 under stress conditions such as heat shock or in the presence of anticancer agents, which might induce heat shock response through the activation of HSF1. Several anticancer agents including etoposide, paclitaxel, carboplatin, and melphalan have been shown to increase HSP expression, especially on the surface of exosome in hepatocarcinoma or multiple myeloma cells [17,18]. Moreover, HSF1 has been shown be involved in the regulation of a specific ABC transporter; its expression is associated with resistance to chemotherapeutic agents [19,20,21]. Thus, the sensitizing effect of KRIBB11 on cancer cells upon anticancer drug treatment can be partially attributed to the accumulation of anticancer drugs in the cytoplasm via suppression of the efflux transporter expression. In contrast, in the absence of stress conditions, KRIBB11 might activate another route to induce apoptosis, such as stabilization of MULE, rather than interference of HSF1 activity. However, considering that non-classical targets of HSF1 have been implicated in various oncogenic properties of HSF1 [22,23,24], and that some targets of HSF1 do not contain the HSE sequences in their promoter regions [25,26], the involvement of HSF1 activity in KRIBB11-induced apoptosis remains to be resolved through further studies by focusing on various effector molecules of HSF1 in the extended lists of cancer cells.

Additionally, our results revealed that BCL-2 expression was decreased at the protein level by KRIBB11. However, BCL-2 levels are not affected by MG132 or MULE (Figure 2 and Figure 3), which indicates that the effect of KRIBB11 on BCL-2 might be different from that on MCL-1. As far as BCL-2 degradation is concerned, it has been shown that dephosphorylation at specific threonine or serine residues on BCL-2 protein facilitates its proteasome-dependent degradation [27,28]. Furthermore, XAIP or Keap1/Cul3 ubiquitin ligase are preferential ubiquitin ligases for BCL-2 rather than MULE [29,30]. Thus, the investigation of KRIBB11 on BCL-2 expression, especially with respect to the alteration of phosphorylation status, should be conducted to clarify the role of BCL-2 in KRIBB11-mediated apoptosis.

Currently, the mechanism by which KRIBB11 stabilizes MULE remains unclear, and this could be regarded as a limitation of our study. Previously, KRIBB11 was shown to associate with HSF1 in vitro [6], and HSF1 protein levels, but not mRNA levels were slightly but definitely decreased following KRIBB11 treatment in our study (Figure 3 and Figure S1). Thus, it is probable that the chemical structure of KRIBB11 might favor interactions with specific proteins by stably fitting into the three-dimensional assembly of the target proteins; their modification could either retard or accelerate the degradation of the binding partners. However, currently, we lack evidence that supports the specific interaction between MULE and KRIBB11. The involvement of interactions of KRIBB11, MULE, or other specific proteins in the KRIBB11-driven cell death should be examined in future studies by labeling KRIBB11 with appropriate tags and subsequently performing Western blotting analysis of the candidate proteins.

In conclusion, we have demonstrated the significant anti-apoptotic effect of KRIBB11 on glioblastoma cells, which is associated with MULE-dependent destabilization of MCL-1. This supports the potential value of KRIBB11 as an anticancer agent against glioblastoma. Our data also highlighted the necessity to study various molecular pathways driven by KRIBB11 in addition to the regulation of HSF1 activity.

## 4. Materials and Methods

### 4.1. Cell Culture and Reagents

A172 human glioblastoma cells were maintained in RPMI 1640 medium supplemented with 10% fetal bovine serum (GeneDEPOT Barker, TX, USA) and penicillin/streptomycin (Biowest, Nuaillé, France) at 37 °C in a humidified incubator with 5% CO_2_. KRIBB11, crystal violet, MG132, and CHX were purchased from Sigma-Aldrich (St. Louis, MO, USA). PI was obtained from Biobud (Seongnam, Korea). The caspase inhibitor Z-VAD-FMK was obtained from Promega (Madison, WI, USA).

### 4.2. Cell Proliferation and Cell Death Analyses

A172 cells were seeded in a 48-well plate at a density of 1.5 × 10^3^ cells/well. The next day, cells were treated with various concentrations of KRIBB11. After 24, 48, and 72 h, 3-(4, 5-methylthiazol-2-yl)-2, 5-diphenyl-tetrazolium bromide (MTT, Sigma-Aldrich) was added (50 μL/well) for 2 h and formazan crystals were solubilized by the addition of acid-isopropyl alcohol. The absorbance of the resulting formazan was measured spectrophotometrically at 570 nm using an ELISA reader (Bio-Rad, Hercules, CA, USA). The relative cell viability was determined as a percentage of the absorbance of treated cells with respect to that of the untreated control cells. For the colony formation assay, A172 cells were seeded into 6-well plates at the density of 1 × 10^3^ cells/well. Post 24 h upon seeding, the cells were treated with KRIBB11 for 2 days and then cultured in a normal medium without KRIBB11 for 13 days, followed by staining with crystal violet. To determine the proportion of dead cells, PI staining and subsequent flow cytometry was performed for A172 cells treated with KRIBB11 for 48 h

### 4.3. Small Interfering RNA (siRNA) Transfection and Quantitative Real-Time PCR (qRT-PCR)

siRNAs targeting HSF1, MULE, CHIP, or MCL-1 were synthesized by Bioneer (Daejeon, Korea) and transfected into A172 cells using G-fectin (Genolution, Seoul, Korea). The sequences of siRNAs are as follows: HSF1, 5′-CUGAAGAGUGAAGACAUAAAGA-3′; MULE, 5′-AAUUGCUAUGUCUCUGGGACA-3′; CHIP, 5′-CGAGCGCGCAGGAGCUCAA-3′; MCL-1, 5′-CAGAACGAAUUGAUGUGUA-3′. RNA was extracted using RNAiso Plus (Takara Bio, Shiga, Japan), and cDNA was prepared using Accupower^®^ Customized Rocket-Script^TM^ Cycle RT premix (Bioneer). To determine the knockdown efficacy of each siRNA transfection or alteration in the indicated mRNA levels, the qRT-PCR was performed using SYBR premix Ex Taq (Takara Bio) on an Applied Biosystems 7300 sequence detection system (Bio-Rad). The sequence of primers used are listed below: MULE, 5′-ACAACCTCGAGCAGCAGCGG-3′ (F) and 5′-TTGTTAGCCCGGCGCGTGTC-3′ (R); HSP70, 5′-CGGGGTAACCGACCAATCAATCTGAAGCCATCT-3′ (F) and 5′-CCGCTCGAGCCGGTTCCCTGCTCTCTGTC-3′ (R); BIS, 5′-AGCCCTCAGCACTGCCCCTGCAGAA–3′ (F) and 5′-GCAGCTCTTTGGTCAAATACTCTTC-3′ (R); HSP27, 5′-TGACGGTCAAGACCAAGGAT-3′ (F) and 5′-ATGGTGATCTCGTTGGACTG-3′ (R); BCL-2, 5′-TGCACCTGACGCCCTTCAC-3′ (F) and 5′-AGACAGCCAGGAGAAATCAAACAG-3′ (R); MCL-1, 5′-CGACGGCGTAACAAACT-3′ (F) and 5′-GGAAGAACTCCACAAACCC-3′ (R); HPRT-1 (Hypoxanthine phosphoribosyltrasferase-1), 5′-TGACACTGGCAAAACAATGCA-3′ (F) and 5′-GGTCCTTTTCACCAGCAAGCT-3′ (R).

### 4.4. Western Blot

Cells were lysed in RIPA buffer (150 mM NaCl, 1% NP 40, 0.5% sodium deoxycholate, 0.1% SDS, and 50 mM Tris-HCL pH 8.0) in the presence of a protease inhibitor (Roche Diagnostics, Mannheim, Germany) on ice for 30 min. Equal amounts of proteins were separated by using 10% sodium dodecyl sulfate-polyacrylamide gel electrophoresis, and then transferred to nitrocellulose membranes (GE Healthcare Life Sciences, Chicago, IL, USA). After transfer, for blocking the membrane, 5% skimmed milk was used in Tris-buffered saline along with 0.1% Tween 20 for 1 h. The membranes were incubated with the primary antibodies for overnight at 4 °C. In this study the following primary antibodies were used: anti-HSF1 (Enzo Life Science, Farmingdale, NY, USA), anti-β-ACTIN (Sigma-Aldrich), anti-cleaved PARP (Abcam, Cambridge, UK), anti-BIS [31], anti-HSP70i (Enzo Life Science), anti-MCL-1 (Cell Signaling, Dallas, TX, USA), and anti-MULE (Abcam). Antibodies against BCL-2, BCL-xL, BAX, and HSP27 were provided from Santa Cruz Biotechnology (Santa Cruz, CA, USA). After incubation with horseradish peroxidase-conjugated anti-rabbit or anti-mouse IgG (1:5000; Santa Cruz Biotechnology), the immunoreactive bands were visualized by using an enhanced chemiluminescence substrate (Thermo Fisher Scientific, Waltham, MA, USA). The intensity of each band was quantified using Image J software (NIH, Bethesda, MD, USA).

### 4.5. Statistics

The data are presented as the mean ± standard error of the mean (SE). Statistical significance between two groups was analyzed by Student’s *t*-test. A *p*-value of <0.05 was considered as statistically significant.

## Data Availability

The data presented in this study are available on the request from the corresponding author.

## Supplementary Materials

The following are available online. Figure S1: Effect of caspase inhibition on the decrease of MCL-1, BCL-2 and HSF1 protein levels induced by KRIBB11.

## Sample Availability

Not available.
