# Supplementary material for: KRIBB11 Induces Apoptosis in A172 Glioblastoma Cells via MULE-Dependent Degradation of MCL-1
Source: Molecules. 2021 Jul 8;26(14):4165. doi: 10.3390/molecules26144165 (PMC8305965; doi:10.3390/molecules26144165)
Supplement: Supplementary file 1 [file molecules-26-04165-s001.zip › molecules-1254699-supplementary.pdf]

molecules

MDPI

Supplementary Materials

KRIBB11 Induces Apoptosis in A172 Glioblastoma Cells via MULE-Dependent Degradation of MCL-1

Kyunghyun Yoo [1,2,3], Hye-Hyeon Yun [1,2], Soon-Young Jung [1,2] and Jeong-Hwa Lee [1,2,*]

1 Department of Biochemistry, College of Medicine, The Catholic University of Korea, Seoul 16591, Korea; ted13579@hanmail.net (K.Y.); nice1205@hanmail.net (H.-H.Y); syjjeong@hanmail.net (S.-Y.J.)
2 Institute for Aging and Metabolic Diseases, College of Medicine, The Catholic University of Korea, Seoul 16591, Korea
3 Department of Biomedicine & Health Sciences, Graduate School, College of Medicine, The Catholic University of Korea, Seoul 16591, Korea
* Correspondence: leejh@catholic.ac.kr; Tel.: +82-2-2258-7293

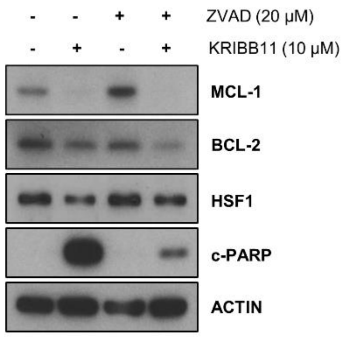

Figure S1. Effect of caspase inhibition on the decrease of MCL-1, BCL-2 and HSF1 protein levels induced by KRIBB11. To inhibit caspase activation, pan-caspase inhibitor Z-VAD-FMK (20 μM) was pretreated to A172 cells 4 h prior to exposure to KRIBB11 (10 μM). After 24 h, western blotting was performed with the indicated antibodies.
